# Implementation of Interprofessional Pharmaceutical Care Initiatives: Lessons Learned from Successful Bottom-Up Initiatives in Primary Care

**DOI:** 10.5334/ijic.7581

**Published:** 2024-04-09

**Authors:** Indira Coenen, Elyne De Baetselier, Veerle Foulon, Tinne Dilles

**Affiliations:** 1 University of Antwerp, NuPhaC, Centre for Research and Innovation in Care, Faculty of Medicine and Health Sciences, Antwerp, Belgium; 2 KU Leuven, Department of Pharmaceutical and Pharmacological Sciences, Clinical Pharmacology and Pharmacotherapy, Leuven, Belgium

**Keywords:** polypharmacy, older people, interprofessional collaboration, pharmaceutical care

## Abstract

**Introduction:**

Although there is evidence that interprofessional, person-centred, integrated care is important for optimising pharmaceutical care of older people with polypharmacy, this way of working is often not implemented in practice. The aim of this study was to identify common characteristics of successful interprofessional initiatives and factors influencing their implementation, in order to close this know-do gap.

**Methods:**

A qualitative, explorative design with in-depth semi-structured interviews was used. Flemish primary healthcare professionals (HCPs) and patients aged over 75, involved in successful initiatives of interprofessional pharmaceutical care for older people with polypharmacy, were included. Inductive analysis was conducted to identify main topics.

**Results:**

Fifteen HCPs and four patients, involved in nine interprofessional initiatives, were interviewed. In all initiatives the HCPs had interprofessional consultations about older people with polypharmacy. The interaction between the characteristics of the initiatives and the context had an important impact on the implementation. These context factors were positioned under the micro-, meso- and macro context. Implementation strategies, actions to enhance the initiatives’ adoption, corresponded with three themes: communication and influence, coordination by different stakeholders, and (dis)incentives.

**Conclusion:**

The identification of these success factors might inspire HCPs, providers of interprofessional education and policymakers to facilitate interprofessional pharmaceutical care.

## Introduction

The World Health Organisation predicts that the proportion of people aged over 60 years will almost double from 12% to 22% in the period from 2015 to 2060 [1]. This demographic shift is called population ageing. People live longer thanks in part to advances in science and the development of better medications. The downside is that people are developing more co-morbidities, requiring them to take more medications. Taking five or more chronic medications is the most commonly used definition of polypharmacy in the literature [2,3].

Pharmaceutical care for polypharmacy patients is often complex and challenging for healthcare professionals (HCPs) due to numerous HCPs across a variety of settings involved, poor communication between HCPs and lack of guidelines to support polypharmacy and multi-morbidity [4,5]. Interprofessional collaboration (IC) plays a crucial role in patient safety [6,7]. For example, ineffective team communication is a contributing factor to medication errors [8,9]. These preventable incidents occur at unacceptably high rates in all healthcare settings [10,11,12]. Lack of effective communication and IC can lead to delays in diagnosis or treatment, which may result in the progression of diseases, worsening of symptoms, and reduced quality of care. This includes increased psychosocial distress and dissatisfaction for patients due to miscommunication between HCPs and a lack of consequent advise from all HCPs involved. Furthermore inefficient IC leads to increased healthcare costs [13,14,15].

IC involves more than just bringing together different HCPs, each applying their unique skills and knowledge in the pharmaceutical care of patients. Indeed, it occurs when two or more professions have mutual respect for each other and each other’s profession and are willing to participate in cooperative working environments to achieve common goals [16,17]. In IC different professions have shared goals in the light of patient outcomes [18,19].

This interprofessional pharmaceutical care for patients with polypharmacy is more successful when it is person-centred and integrated. In person-centred care, it is important HCPs approach patients as unique persons and organise healthcare around the health needs and expectations of the person rather than around diseases [20]. The impact of person-centred care on improving patient outcomes – such as quality of life – and reducing costs, has been previously demonstrated [21]. Integrated care is often used as a synonym for terms such as coordinated care and seamless care. However, there are several definitions of ‘integrated care’, probably resulting from “the polymorphic nature of integrated care itself” [22]. A systematic review of the effect of integrated care, published by Baxter et al, concluded that integrated care may enhance patient satisfaction, increase perceived care quality, and enable access to services, although the evidence for other outcomes including service costs remains unclear [23].

Despite the advantages shown, the level of implementation of interprofessional, person-centred, integrated pharmaceutical care for older people with polypharmacy remains low in Flanders (Belgium). A systematic review of the evidence-practice gap recommended that individuals, wishing to implement any type of change in their organisation, should consider and describe the context they are working in; and need to monitor this context periodically as it is likely to change over time [24]. In this study we searched for successful bottom-up interprofessional, person-centred, integrated initiatives for older people with polypharmacy. From these initiatives, this study aimed to identify a) intervention characteristics; b) factors influencing their implementation, including context factors and implementation strategies, and c) experiences from older people involved in these initiatives.

## Methods

### Design

This study had a qualitative, explorative design. The aim of this study was to identify good practices of IC for older people with polypharmacy. Care for older people is often framed in a negative way. The interdisciplinary research team aimed to inspire other HCPs and policymakers by bringing a positive story about care for older people. After identification and description of successful examples of interprofessional, person-centred, integrated pharmaceutical care for older people with polypharmacy, in-depth semi-structured interviews were conducted with HCPs and older people involved in these initiatives, to identify aspects that influenced the implementation of these initiatives.

All key components of a qualitative study were reported in accordance with the COREQ checklist, this can be found in the Supplementary Data. V.F., T.D. and E.D.B. are senior researchers with extensive experience in qualitative research. They worked intensively with junior researcher I.C.

### Participants

In April 2022, a call was launched via different channels (social media and email) to identify successful Flemish primary healthcare initiatives where collaboration between at least two HCP groups existed (convenience sample). Various professional associations for nurses, pharmacists and doctors; umbrella organisations and pilot projects of integrated care in Flanders were contacted via email to disseminate the questionnaire. In addition, a call was launched in the researchers’ professional networks. The interprofessional initiatives had to be related to pharmaceutical care for older people with polypharmacy. Polypharmacy in older people was defined as the use of at least five medicines a day in patients aged over 75. Both small-scaled bottom-up initiatives and larger coordinated initiatives were considered. An initiative was defined “successful” when it was already implemented in practice, and the involved HCPs perceived it as a good and inspiring example of IC for other teams. Three key elements for successfulness were asked at the call for participation and evaluated by two authors (I.C. and E.D.B.) before enrolment in the study.

The call included a link to a short online questionnaire. This questionnaire surveyed inclusion criteria, characteristics of the initiative and asked for contact details of potential reference HCPs to be interviewed.

Patients, who were involved in these initiatives, were selected after the interviews with HCPs. Contact details of patients over the age of 75, who took at least five medicines a day, were provided by the HCPs after patients’ permission.

Recruitment of participants was performed until data sufficiency, i.e. two independent researchers confirmed no added information was identified after analysing the last two interviews.

### Data collection

Single interviews were conducted either with one HCP (n = 5) or two HCPs (n = 5) by I.C. and E.D.B. (both female pharmacist and nurse, respectively). Interviews with HCPs were done online or live in the healthcare setting; interviews with patients took place at the patient’s home or in the nursing home. Besides the participants and the researchers, there was no one else present during the interviews.

The process of triangulation was applied first in the data collection through recruiting participants from different healthcare professions and their patients. Second, the researchers who conducted the interviews and then analysed them independently, have diverse backgrounds (pharmacy and nursing). Based on a semi-structured interview guide, HCPs were questioned about the organisational and motivational aspects of the initiatives. The content of the interview guide was agreed after discussion with the interdisciplinary research team, clarity was evaluated using a pilot interview. This included the ideas and inspiration at the basis of the initiative and factors influencing the initiative’s implementation. In the patient interviews, perceived added value for the patient, positive aspects and potential obstacles were addressed. All interviews were audio recorded with a smartphone (live interviews) or through Microsoft Teams (online interviews). The interview guides of HCPs’ en patients’ interviews, can be found in the Supplementary Data.

### Data analysis

The interviews were transcribed *ad verbatim* to facilitate reflexive thematic data analysis. To increase the reliability of the data analysis, interviews were coded independently by the two aforementioned researchers. Differences in codes were discussed until consensus. In one interview, the researchers did not reach consensus, therefore member checking was performed to improve accuracy, credibility and validity of the results. During the member checking, the researchers mailed the interview transcript to the participant and asked to review and clarify a particular paragraph.

Initially, the analysis of the results was conducted inductively to identify codes and themes emerging from the transcripts. In a following phase, to structure the results, the themes were compared with and positioned under the core elements of determinant frameworks, identified by Huybrechts et al [25]. This study identified the core building blocks of existing implementation frameworks and models, which can be used as a basis to further develop a framework for the implementation of complex interventions within primary care practices. These core components, identified as common elements of most implementation frameworks, were: intended change, context and implementation strategies.

### Ethical considerations

This study was approved by the Ethics Committee UZA/UA (registration number BUN B3002022000039). All participants received oral and written information and gave written consent before starting the interviews. Personal data were processed pseudonymised. Participants could withdraw their consent up until the point of data analysis, without giving any reasons.

## Results

### Interprofessional initiatives in the pharmaceutical care for older people with polypharmacy in primary care in Flanders

In total, 20 respondents answered our call for information about interprofessional initiatives in the care for older people with polypharmacy. Within these responses, 16 unique initiatives could be identified of which 10 corresponded to the inclusion criteria and were eligible for further exploration. Table 1 provides an overview of the initiatives included. In total, 15 HCPs of 9 different initiatives were interviewed. Five of these initiatives were situated in ambulatory healthcare – care patients receive at home – and four in the nursing home setting. The interviewees were seven project coordinators (nurse, pharmacist, speech therapist, occupational therapist or social worker), two (head)nurses, one pharmacist, one geriatrician, one physiotherapist, one nursing home director and two general practitioners (GPs). Both GPs also served as coordinating and advising physicians in a nursing home, coordinating the medical care provided by the independent, visiting GPs of the residents. The patient interviews (n = 4) were equally spread over home care patients and nursing home patients.

**Table 1:** Overview of included Flemish interprofessional initiatives for older people with polypharmacy in primary healthcare.


INITIATIVE	SETTING	DESCRIPTION	ROLE OF INCLUDED HEALTHCARE PROFESSIONALS

1	Ambulatory healthcare	Therapy adherence screener, medication review	The **nurse** or **pharmacist** evaluated the patient’s therapy adherence by using a therapy adherence screener to guide the conversation with the patient. Next, the completed screener and the medication schedule were provided to the **general practitioner** (GP) and pharmacist. The GP and pharmacist conducted a medication review together. Patient education was then provided by the pharmacist, GP or nurse.

2	Ambulatory healthcare	Proactive monitoring of (poly)medication	The **nurse** had a conversation with patient to monitor the medication use. Areas of concern were discussed with the **GP**, who provided a follow-up meeting with the patient.

3	Ambulatory healthcare	Individual medication preparation, medication review	The **nurse** had a conversation with patient to monitor the medication use and identified patient who would benefit from individual medication preparation. The **GP** and **pharmacist** conducted a medication review together. The pharmacist then conducted the individual medication preparation at the community pharmacy.

4	Ambulatory healthcare	Multidisciplinary ‘neighbourhood teams’	The **nurse, pharmacist, GP, physiotherapist** and **psychologist**, who work in the same area, had interprofessional meetings to make agreements to optimise pharmaceutical care for specific patient populations (e.g. patients with heart failure).

5	Ambulatory healthcare	Individual medication preparation, medication review	The **nurse** or **pharmacist** evaluated the patient’s medication use. The **GP** and pharmacist conducted a medication review together. The pharmacist then conducted the individual medication preparation at the community pharmacy and provided patient education.

6	Nursing home	Interprofessional case conferences	A structured interprofessional case conferences was organised (twice a year) with the **nurse, pharmacist, GP** and **coordinating physician** to discuss residents’ medication schedules

7	Nursing home	Interprofessional case conferences	A structured interprofessional case conferences was organised (thrice a month) with the **nurse, pharmacist, GP, coordinating physician, occupational therapist** and **physiotherapist** to discuss residents’ medication schedules. The medication review was prepared by the pharmacist before the case conference.

8	Nursing home	Interprofessional case conferences	A structured interprofessional case conferences was organised (once in three weeks) with the **nurse, general practitioner, nurse aid, occupational therapist, physiotherapist** and **nursing home director** to discuss residents’ medication schedules.

9	Nursing home	Periodic evaluation of psychotropic drug use	A structured interprofessional meeting was organised (thrice a year) with the **nurse, pharmacist, general practitioner, coordinating physician, psychologist** and **nurse aid**. This meeting was prepared by the psychologist who monitored psychotropic drug use and medical records of residents.


In ambulatory healthcare, the interprofessional initiatives consisted of a therapy adherence screener, proactive monitoring of polypharmacy, individual medication preparation, multidisciplinary neighbourhood teams and guidance on proper use of Parkinson’s medicines. In nursing homes three initiatives about interprofessional case conferences and one initiative about periodic evaluation of psychotropic drug use were explored.

### Factors influencing the implementation of the initiatives

**Figure 1:**
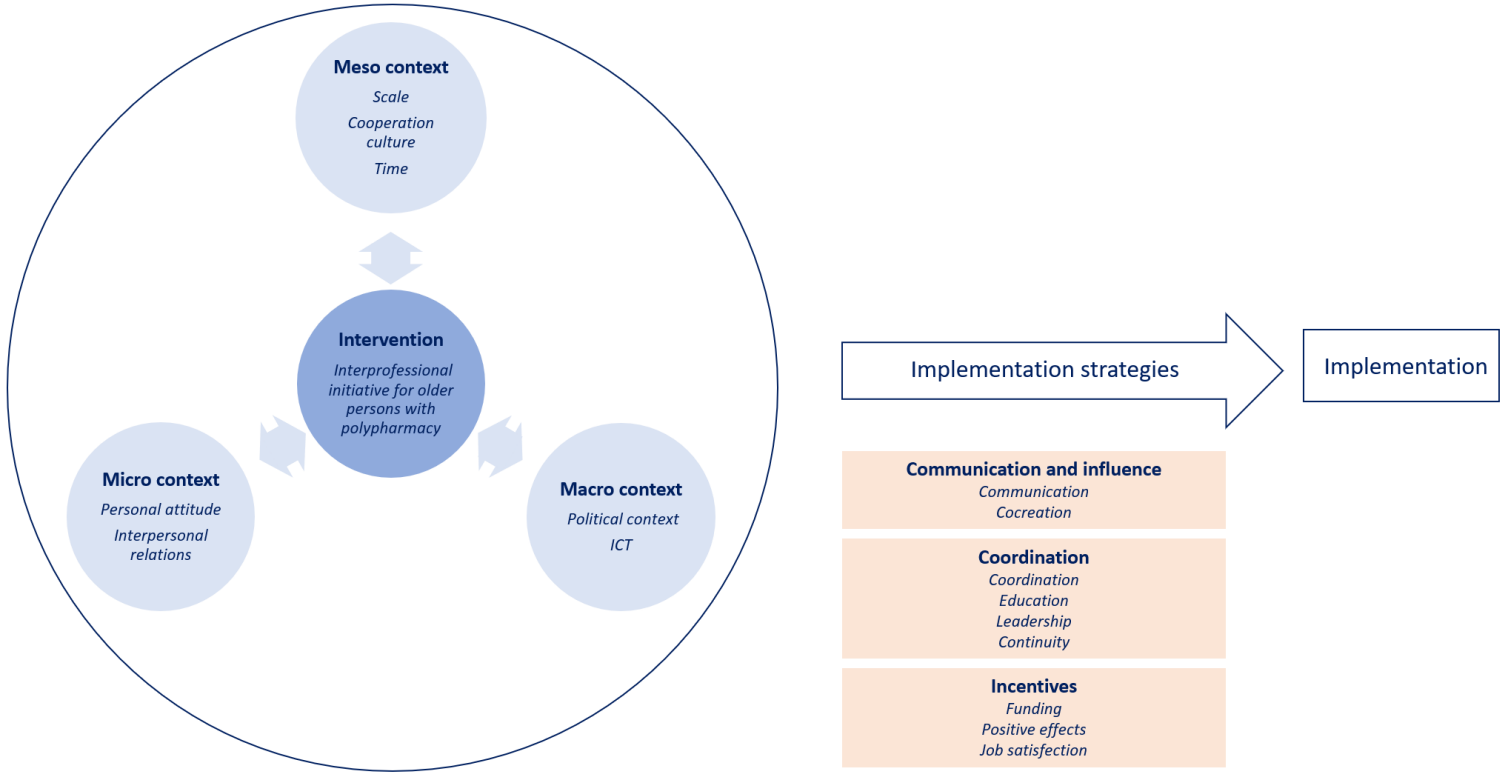
**Themes** and *subthemes* influencing implementation of interprofessional initiatives for older people with polypharmacy positioned under the three core elements – intended change (dark blue), context (light blue) and implementation strategies (orange) – of implementation frameworks

The data analysis identified common elements between the successful initiatives. Figure 1 shows the seven themes and seventeen subthemes identified from the interviews, positioned under the three core elements (intended change, context and implementation strategies) that have been identified in earlier research upon analysis of commonly used implementation frameworks [25].

In the centre of the figure, we positioned the common characteristics of the initiatives as they represent the changes that were implemented to achieve higher quality interprofessional pharmaceutical care. In implementation frameworks, this is commonly referred to as ‘the intended change’ [25].

Context variables can be defined as “the set of circumstances or unique factors that surround a particular implementation effort” [26]. This context played a dynamic role and included three levels: a) the micro context with influences of HCPs’ personal characteristics and interpersonal relationships, b) the meso context, including the influence of the scale of the initiative, collaboration culture and time available, and c) the macro context of the initiative which included the role of the government and availability of ICT infrastructure.

Whereas the intended change refers to what is to be implemented, the implementation strategies refer to how they have to be implemented and are linked to the process or mechanism that intervention designers want to trigger to accomplish implementation.^25^ Different implementation strategies were identified corresponding with three themes: communication and influence; coordination of different stakeholders; and incentives and disincentives.

### Intended change – intervention characteristics

All initiatives had a bottom-up design with voluntary HCP participation. The initiatives aimed to optimise the pharmacotherapy of older people with polypharmacy via interprofessional consultations. HCPs mentioned they had mutual respect and appreciation for other HCPs’ input in these consultations and team-made decisions. This was said to result in better interprofessional communication and collaboration. With the intention to improve the quality of care, HCPs aimed for a holistic approach combining the information and perspectives of the different HCPs with the expectations and needs of the older people and/or their carers. This resulted in more integrated, proactive and safe pharmaceutical care. Interviewees hoped that the initiatives would serve as an example of a ‘Good Practice’ to inspire other HCPs. For the nursing home setting one participant mentioned that the initiative enabled a smooth transfer from the home setting to the nursing home and the initiative empowered nursing home residents.

### Context – circumstances that surround an intervention

**On a micro level**

*Personal motivation and attitude as a starting point*

All participants mentioned that a respectful, enthusiastic and open attitude facilitated IC. Key intrinsic motivators for HCPs were the realisation that a) HCPs care for the same older people which connects them, b) IC improves the quality of care for older people with polypharmacy and thus can be considered as a societal responsibility, and c) participation in the initiative provides an opportunity for further learning and professionalisation of HCPs. Additionally, previous experience with IC (e.g. in preparatory education) was identified to enhance cooperation willingness. Resistance to change (e.g. the tendency to want to protect one’s own professional territory) was frequently observed and hindered collaboration. (quote, Table 2)

**Table 2:** Quotes illustrating different subthemes related to context factors.


SUBTHEME	QUOTE	INTERVIEWEE	INITIATIVE

Personal characteristics	*In the beginning there was some resistance of our nurses to hand over the task of medication preparation. Or it could be fear to no longer being fully up-to-date*.	Nurse	3

Interpersonal relations	*Now they simply know us and our capacities better. There is respect and awareness that we do have medication knowledge. Without IC, this would not be clear*.	Pharmacist	7

Culture of cooperation and intervision	*“It should be interprofessional and so they should all be under the same roof”, this is nonsense. There should be a collaborative culture, you should sit together but it is not necessary to work under one roof*.	General practitioner	7

Time creates opportunities	*The pharmacists, we collaborate with, are often small scaled pharmacists, who have no time to invest in such collaboration initiatives about polypharmacy. This should be supported much better*.	Nurse	2

Political context	*If you were to aggregate the profit of this initiative, it should actually be mandatory. (…) As a GP, you should take it for granted that someone is double-checking your prescriptions. It is so obvious*.	Nursing home director	6

Restricted ICT possibilities	*I think the integration of the medical electronic record and the nursing electronic record is a priority to be able to avoid misunderstandings and interactions between medication in particular. (…) It would not solve all the problems but it would make it a lot easier in terms of communication between the different actors in the nursing home*.	Coordinating physician	7


**Interpersonal relations – the importance of building trust**

Face-to-face interaction and transparency (e.g. on different HCPs’ roles), while discussing the pharmacotherapy of older people, were perceived as important facilitators to build mutual trust between HCPs. Perceiving other HCPs as equals and valuing their competences enabled IC. Informal contacts were mentioned as an efficient way to enhance the cohesion of the healthcare team and lower the threshold for IC. In contrast, high staff turn-over of nurses and absenteeism from meetings were described as barriers. All interviewees mentioned that the GP has the last word on the pharmacotherapy of the older people; hence, difficult accessibility and absenteeism of GPs at an interprofessional case conference were experienced as delaying factors for making improvements in pharmacotherapy of people with polypharmacy. Moreover, patients have the right to choose each individual HCP in primary care. This results in the fact that HCPs must collaborate with a lot of HCPs they barely know. This freedom of choice regarding HCPs in primary care was mentioned as a complicating factor for IC. (quotes, Table 2)

**On a meso level**

*The scale of the interprofessional network matters*

HCPs indicated that a small-scaled network, with close collaborations between HCPs, works best in practice. Likewise, initiatives that originate from larger organisations, with managers far removed from clinical practice, were seen as an obstacle to IC.

*Culture of collaboration and intervision*

According to the participants, a culture of intervision facilitated the accessibility and sustainability of IC. Furthermore, it was mentioned that housing different disciplines in one building was not a sufficient condition for IC. If there was no culture of collaboration, it appeared that even in one building HCPs worked alongside and separately from each other. In the context of a nursing home, the presence of a director supporting a collaborative culture was experienced as a major facilitator. (quotes, Table 2)

*Time creates opportunities*

The availability of dedicated time for IC was considered essential. Preparation was deemed necessary to efficiently use the interprofessional time. Although HCPs expressed the importance of holistic care, it was mentioned that it is important to focus on the pharmacotherapy of the patient during interprofessional meetings in order to efficiently use the interprofessional time. High workload was indicated as an important barrier to implement interprofessional initiatives. When not integrated into daily clinical practice, HCPs did not consider the initiative as a priority. (quote, Table 2)

**On a macro level**

*Political context*

The HCPs, who were either part of a temporarily subsidised pilot project or who voluntarily participated in the interprofessional initiative, indicated that financial incentives are necessary for the viability and sustainability. A legal framework that provides transparency on defined roles and honoraria of the various HCPs, was seen as vital for both implementation and larger scale sustainability of the initiatives. (quotes, Table 2)

*Restricted possibilities of ICT*

Electronically sharing patient data in a secure manner was identified as a facilitator. However, the currently available ICT possibilities were considered as limited and hence a potential barrier for IC. In the nursing home setting, a lack of an integrated patient record hindered the information flow. (quote, Table 2)

### Implementation strategies – actions to enhance implementation

**Communication and influence**

*Communication*

Firstly, using multiple communication channels was experienced to raise awareness about the initiative and to motivate HCPs to participate. Professional associations were often involved in this communication. Secondly, communication between HCPs, facilitated by a) (electronically) shared patient data, b) preparation of interprofessional time, c) face-to-face consultation and d) clear agreements between HCPs, was thought to support the implementation of the initiative. (quote, Table 3)

**Table 3:** Quotes illustrating subthemes related to implementation strategies.


SUBTHEME	QUOTE	INTERVIEWEE	INITIATIVE

Communication	*We made a very simple flow chart for three medication classes: benzodiazepines, antidepressants and antipsychotics, presented on a poster visible for the entire staff (…) I think this is very important: translating the evidence into everyday language and no bombastic messages but that everyone could understand and apply it*.	Geriatrician	9

Co-creation	*The inspiration (for the initiative) came from within the organisation. Problems were reported, e.g. difficulties with finding information about medication or to give feedback. We saw that there was too little contact with pharmacists about medication use. Hence, we wanted to set up this collaboration*.	Nurse	3

Coordination	*It is a multi-year journey and there are still things that need to be improved. (…) It is important to realise that starting up a multidisciplinary neighbourhood team like this, you have to approach it very phased and carefully, don’t want to do everything at once. That won’t work*.	Project coordinator	4

Education	*You have to inform and educate all the staff of the nursing home about the different aspects of the medication use. (…) it is very important to get all noses in the same direction*.	Geriatrician	9

Leadership	*Giving leadership to HCPs is incredibly important. (…) That makes it for me and my colleagues, the coordinators, a lot easier. We don’t have ‘to pull’ all the time because there’s so much happening bottom-up*.	Project coordinator	4

Continuity	*You need a fixed point of contact (of the delivering pharmacy) for the continuity of care. It is important to have trust in each other for the sustainability of the interprofessional consultations. What helps is the fact that you work together for a long time*.	Coordinating physician	7

Funding	*Our intention is that hopefully this (initiative) can become something structural and that a nomenclature code can be linked to it in such a way that this becomes the regular operation of the pharmacist*.	Project coordinator	5

Positive effects	*We think this collaboration is very important. As nurses, we look at things differently than doctors. They look from the medical and we rather from the practical side. We notice different things. Bringing those two stories together makes it much easier to follow-up someone*.	Nurse	2

Increased job satisfaction	*I think everyone feels more appreciated. We work together with respect for each other and for each discipline. I would be very frustrated if I had to do my job without being able to collaborate, and if we would work on separate islands. I would be less motivated to work. So I think that (initiative) stops or removes a lot of internal frustrations*.	Physiotherapist	8


*Co-creation with HCPs*

All initiatives had a bottom-up design and originated from a daily practice need experienced by HCPs. Having experienced some form of co-creation, i.e. HCPs having been involved in the start-up of the initiative, was indicated as an important facilitator for implementation. In addition, project coordinators indicated that regular intervision with HCPs was an efficient way to stay in contact with the professional field. (quotes, Table 3)

**Coordination**

*Coordination of the initiative*

The initiatives that were explored, were coordinated by the HCPs themselves or by project coordinators of larger pilot projects. An initially well-structured organisation of the initiative and a gradual implementation were identified as important factors for the implementation. According to the interviewees, professional associations can have a supportive role in a structured implementation of interprofessional initiatives, e.g. by reducing the administrative burden in requesting funding. In addition, it was mentioned that compact documentation can be supportive for the implementation of the initiatives. (quotes, Table 3)

*Education*

Training for HCPs to enrol in the initiative was identified as a facilitator for adequate implementation. Inspiration for training material was drawn from ‘good practices’ emerging from other initiatives. Comprehensive training ensured HCPs being aligned and accelerated the implementation of the initiative. In addition, interviewees expressed the importance of translating scientific expertise into clinical practice guidelines to facilitate implementation. (quote, Table 3)

*Leadership*

In most initiatives, a project coordinator was necessary to support HCPs to initiate the initiative. However, the importance of HCPs to become self-directed for the sustainability of the initiative was emphasised. The presence of an inspirational leader or a steering group was an important facilitator for the implementation. Moreover, specifically in the nursing home setting, the importance of an engaged management team was highlighted. (quote, Table 3)

*Continuity*

Continuity in collaboration was perceived as a facilitating factor. As mentioned, high staff turn-over was experienced as an inhibitor of IC as it makes formation of cohesive teams more difficult. In addition, pro-active and regular scheduling of interprofessional meetings were perceived important to the sustainability of the initiative. (quote, Table 3)

**Incentives**

*Funding – enabling sustainability and appreciation*

As mentioned above, political support was seen as an important macro-contextual factor affecting financial feasibility of the initiatives. According to the interviewees, initial grants made it possible to test workflows and establish good practices. However, great uncertainty regarding the initiatives’ funding was experienced resulting in doubts about sustainability. In addition, interviewees were convinced that financial compensation for collaborating HCPs could lead to IC becoming a part of standard care. Some participants indicated this is especially important for pharmacists because they mainly receive payments per delivered medication, and their role in the initiatives falls outside the scope of standard pharmaceutical care. Additionally, interviewees mentioned that a (small) financial incentive can provide an experience of appreciation for the care provided by pharmacists. (quote, Table 3)

*Positive effects of the initiative*

The visibility of good practices was indicated as a facilitator for HCPs to start the initiative. Similarly, revealing (intermediate) results and patient appreciation are important motivators to sustain HCPs’ efforts. Other motivating factors mentioned were HCPs’ awareness of a) complementarity of different professionals, b) benefits of having a more critical attitude and c) being able to provide more qualitative care by collaborating. As the positive effects of IC were evident to the interviewees, IC was considered as self-evident and a societal responsibility. (quote, Table 3)

*Increased job satisfaction*

By participating in interprofessional consultations, interviewees felt more valued in their role as HCP. Furthermore, belonging to a group was perceived positively and shared responsibility was mentioned to reduce stress. As a result, these factors increased HCPs’ job satisfaction. (quote, Table 3)

### Limited feedback of older people

**Feedback of older people on the received pharmaceutical care**

Interviews with older people were significantly shorter than those with HCPs (approximately 30 minutes versus 60 minutes). The four patients mentioned they were satisfied with the pharmaceutical care they received and none of them had improvement suggestions on the HCPs’ approach. Furthermore, the interviews demonstrated that older people appreciated continuity, accessibility, regular follow-up of their health status (including medications), proximity, attitude of respect, transparent and clear communication, decisiveness, helpfulness and HCPs with job satisfaction.

**Limited awareness of IC**

All initiatives, that were explored through the HCP interviews, were examples of person-centred care, at least in their ambitions. This means that changes in pharmacotherapy were tailored to the health needs of the older people. However, the HCP interviews showed that little feedback was requested from the older people about the initiatives. HCPs mentioned two reasons why there was no direct patient communication about IC. On the one hand, the IC was taken for granted by older people and on the other hand older people seemed not to have information needs in this regard.

Indeed, the four interviews with older people confirmed that these patients were not aware of interprofessional initiatives, and its HCPs involved. Two older people mentioned the GP was the only HCP involved in pharmacotherapy. When the researchers explained the interprofessional initiatives, all interviewed older people appreciated these. The four interviewed patients mentioned they felt no need to be present at interprofessional medication consultations because they trusted the decisions of HCPs. Two older people mentioned there was sufficient communication with patients (or with their carers) if changes in their pharmacotherapy occurred. The two other older people expressed to have no need for information in case of medication changes.

“My medication is organised by the doctor. If there is something to discuss, the nurse books an appointment with the doctor. My medication is not discussed with the nurse. The nurse checks what medication that I take and asks what I need from the pharmacist. Then I go to the pharmacist.” Patient 1, initiative 2.

## Discussion

This study was set up to help close the gap between what is known to be beneficial for quality of pharmaceutical care and common clinical practice. The aim was to learn from successful initiatives in interprofessional, person-centred, integrated care. Common characteristics of initiatives and factors influencing their implementation were identified.

Several domains that influence the implementation were analysed: the intervention characteristics, the organisational context on a micro, meso and macro level and specific implementation strategies. In each of these domains more specific barriers and facilitators have been identified to be considered when setting up similar initiatives.

All initiatives had a bottom-up approach, illustrating the importance of co-creation, with voluntary participation of HCPs. HCPs considered the initiative as an extra task on top of standard care. Surprisingly though, this is not how patients perceived it. They did not seem to be aware of the initiative. They gave positive feedback about care received, but did not perceive it as an additional interprofessional initiative. The HCPs did suggest that – to ensure sustainability – the initiatives should be considered as standard care in the future and should be supported by governments through the development of a legal framework and funding.

To improve interpersonal relations, HCPs suggested to have face-to-face contact on a regular basis and to communicate about each other’s roles. Previous research confirmed that lack of shared expectations of collaboration by community pharmacists and physicians and lack of routine face-to-face interactions are barriers that need to be addressed in the process of medication review [27]. Our study showed that this need for face-to-face interactions was not related to a need to house different disciplines in one building. A recent study even mentioned that GPs who have a ‘very favourable to cooperation’ profile worked less frequently in multi-professional group practices [28].

Traditionally, drug prescription and follow-up have been the sole responsibility of physicians [28]. This was also the opinion of the older people interviewed in this study. Nevertheless, the added value of nurses and pharmacists in pharmaceutical care has been clearly demonstrated [29]. Our results showed that the limited accessibility and the absenteeism of GPs at an interprofessional medication consultation was a delaying factor for making improvements in pharmacotherapy of older people with polypharmacy. Previous research, investigating nurses’ and pharmacists’ learning experiences from participating in interprofessional medication review for older people in primary healthcare, confirmed these findings [30].

In our interviews, only one HCP, who was involved in training GPs, mentioned participating in interprofessional education as a facilitator for IC. This is remarkable since several other studies have described interprofessional education as an important enabler [28,31]. Yet, in the current Flemish context, these results are less surprising because in Flanders no substantial interprofessional education programme exists that focuses on pharmaceutical care with nurses, pharmacists and physicians. There are some initiatives in undergraduate education, but these were only recently set up.

### Implications for clinical practice and future research

The benefits of IC and communication between pharmacists, physicians and nurses and its major impact on care quality and patient outcomes have already been amply demonstrated [32,33]. Yet, healthcare systems are historically hierarchical in nature with physicians regularly assuming leadership positions and decision-making roles [34]. Our results corroborated that mutual respect and appreciation for all HCPs’ input in interprofessional consultations is essential.

Frustrations between HCPs, due to differences in communication styles, and lack of self-confidence or organizational hierarchies hinder interprofessional relationships and communication [34]. To address this, it may be helpful for team members to have regular consultations and to agree on roles and responsibilities. Increasing the awareness of all team members’ potential roles would allow pharmacists, nurses and physicians to benefit from teamwork [32]. Previous research showed that nurses and pharmacists benefit from learning from each other when participating in interprofessional medication reviews [30].

As the positive effects of the initiatives have been clearly demonstrated, we recommend policymakers to support sustainable implementation on a large scale. This is important to provide a transparent legal and financial framework enabling incorporation of regular and structured interprofessional consultations about pharmacotherapy in daily practice.

More in-depth research to explore patients’ views on IC is suggested. This could include investigating patients’ perspectives (including all age groups) on a) IC, b) roles of different HCPs in pharmaceutical care, and c) freedom of choice of different HCPs, compared with choosing a well-aligned team.

### Strengths and limitations

The aim of this study was to provide a comprehensive overview of factors that can impact successful implementation of interprofessional, person-centred, integrated, pharmaceutical care. Our results offer opportunities to inspire HCPs to collaborate interprofessionally in pharmaceutical care for older people with polypharmacy.

The quality of this study can be demonstrated based on the qualitative research criteria of Lincoln and Guba [35]. Firstly, triangulation of sources and analyst triangulation indicate *credibility*. Secondly, the extensive focus on the IC experiences within the pharmaceutical care context of the participants resulted in thick descriptions, which facilitates *transferability* of the study findings. Although our study focused on the specific context of primary healthcare in Flanders, comparing our study results with the European literature confirmed the transferability of several findings. Thirdly, the *dependability* is confirmed by investigator triangulation: coding of all interviews was done by two researchers independently. Fourthly, the *confirmability* of this study is shown by the member checking that has been performed.

This study focused on HCPs’ experiences and recommendations for IC. Therefore, an important limitation of this study was the limited feedback of older patients involved in the initiatives. Although efforts were made to include the patient’s perspective, the results were very limited due to too few patients having been approached by their HCPs to and patients not being aware of interprofessional initiatives.

## Conclusion

Although there is evidence that interprofessional, person-centred, integrated care is important for optimising pharmaceutical care of older people with polypharmacy, this way of working is often not implemented in practice. This project identified and described successful initiatives between physicians, pharmacists and nurses, their context and implementation strategies. The results of this study can be inspirational for HCPs, providers of interprofessional education and policymakers to facilitate interprofessional pharmaceutical care in order to close the evidence-practice gap.

## Additional File

The additional file for this article can be found as follows:

10.5334/ijic.7581.s1 Supplementary Data. Supplementary data 1 to 3.
