# Supplementary material for: Implementation of Interprofessional Pharmaceutical Care Initiatives: Lessons Learned from Successful Bottom-Up Initiatives in Primary Care
Source: Int J Integr Care. 2024 Apr 9;24(2):5. doi: 10.5334/ijic.7581 (PMC11012220; doi:10.5334/ijic.7581)
Supplement: Supplementary Data. — Supplementary data 1 to 3. [file ijic-24-2-7581-s1.pdf]

Supplementary data

Supplementary data 1: Consolidated criteria for reporting qualitative studies (COREQ)

No	Item	Guide questions/description	Response
**Domain 1: Research team and reflexivity**			
Personal Characteristics			
1.	Interviewer/facilitator	Which author/s conducted the interview or focus group?	It is mentioned in the methods (data collection)
2.	Credentials	What were the researcher's credentials? *E.g. PhD, MD*	It is mentioned in the methods (data collection)
3.	Occupation	What was their occupation at the time of the study?	We added the researchers’ affiliations to the authors information
4.	Gender	Was the researcher male or female?	We added the researchers’ gender to the authors information in the method section
5.	Experience and training	What experience or training did the researcher have?	We added the researchers’ experience to the authors information in the method section.
Relationship with participants			
6.	Relationship established	Was a relationship established prior to study commencement?	Yes, we added to the methods that the call was also launched in the researchers’ professional networks.
7.	Participant knowledge of the interviewer	What did the participants know about the researcher? e.*g. personal goals, reasons for doing the research*	At the beginning of each interview, we explained the reasons for doing the research. It is mentioned in the interview guides we added as supplementary data

8.	Interviewer characteristics	What characteristics were reported about the interviewer/facilitator? e.g. *Bias, assumptions, reasons and interests in the research topic*	The researchers' motivation to conduct this study was added to the design section
**Domain 2: study design**			
Theoretical framework			
9.	Methodological orientation and Theory	What methodological orientation was stated to underpin the study? *e.g. grounded theory, discourse analysis, ethnography, phenomenology, content analysis*	The motivation to start te study was added to the design section and the use of a theoretical framework to guide the data analysis, is mentioned in the data analysis section.
Participant selection			
10.	Sampling	How were participants selected? *e.g. purposive, convenience, consecutive, snowball*	*Convenience*<br>It is added to the method section
11.	Method of approach	How were participants approached? e.*g. face-to-face, telephone, mail, email*	*Email*<br>It is mentioned in the method section
12.	Sample size	How many participants were in the study?	19<br>It is mentioned in the result section
13.	Non-participation	How many people refused to participate or dropped out? Reasons?	Not applicable
Setting			
14.	Setting of data collection	Where was the data collected? e.*g. home, clinic, workplace*	*Online, home or nursing home*<br>It is mentioned in the method section
15.	Presence of non-participants	Was anyone else present besides the participants and researchers?	No, we added this information to the method section
16.	Description of sample	What are the important characteristics of the sample? *e.g. demographic data, date*	It is mentioned in the results.<br>For confidentially reasons we just reported the description of the

			initiative and professional activity of participants.
Data collection			
17.	Interview guide	Were questions, prompts, guides provided by the authors? Was it pilot tested?	Yes<br><br>It is added to the method section
18.	Repeat interviews	Were repeat interviews carried out? If yes, how many?	No, we added to the method section single interviews were conducted
19.	Audio/visual recording	Did the research use audio or visual recording to collect the data?	Yes<br><br>It is mentioned in the method section
20.	Field notes	Were field notes made during and/or after the interview or focus group?	No field notes
21.	Duration	What was the duration of the interviews or focus group?	30 minutes (patient interviews) and 60 min (HCP interviews) It is mentioned in the results section
22.	Data saturation	Was data saturation discussed?	Yes<br><br>We added this to the method section
23.	Transcripts returned	Were transcripts returned to participants for comment and/or correction?	Yes<br><br>We added this to the method section
**Domain 3: analysis and findings**			
Data analysis			
24.	Number of data coders	How many data coders coded the data?	We mentioned in the method section two researchers coded independently
25.	Description of the coding tree	Did authors provide a description of the coding tree?	No<br><br>The identified themes and subthemes are presented in Figure 1
26.	Derivation of themes	Were themes identified in advance or derived from the data?	It is mentioned in the method section

			(inductive analysis)
27.	Software	What software, if applicable, was used to manage the data?	NA
28.	Participant checking	Did participants provide feedback on the findings?	Yes<br><br>We added this to the method section
**Reporting**			
29.	Quotations presented	Were participant quotations presented to illustrate the themes / findings? Was each quotation identified? e*.g. participant number*	Yes<br><br>It is mentioned in the results section
30.	Data and findings consistent	Was there consistency between the data presented and the findings?	Yes<br><br>It is mentioned in the results section
31.	Clarity of major themes	Were major themes clearly presented in the findings?	Yes<br><br>It is mentioned in the results section
32.	Clarity of minor themes	Is there a description of diverse cases or discussion of minor themes?	Yes<br><br>The description of the findings is presented based on themes and subthemes

Supplementary data 2: interview guide healthcare professional

The purpose of this interview is to comprehensively delineate your initiative. To start, I would like to ask you to introduce your initiative within a two-minute time limit. To aid in this, it may be helpful to first identify the three key elements.

In what ways does your initiative serve as an exemplar for interprofessional and integrated patient-centered care?

What prompted the initiation of the initiative? What challenges were encountered in pharmaceutical care for older people with polypharmacy?

Where did you draw inspiration for the initiative?

How did the implementation of the initiative unfold?

How is the initiative currently organized?

What roles do the various involved healthcare professionals play?

What are the barriers and facilitators for interdisciplinary collaboration?

What motivates you?

What positive effects have you observed for the healthcare professionals and patients involved?

What ensures the sustainability of the initiative?

What distinguishes your initiative? What key points should be remembered from this interview?

Lastly, what advice would you offer to other healthcare professionals who are still exploring similar initiatives?

Supplementary data 3: Interview guide patient

We came to you via your healthcare professionals, who work together to optimise your medication. The aim of this conversation is to find out how you experience this care and cooperation between your healthcare professionals and how they could support you even better.

How do you experience the collaboration between your various healthcare professionals?

What do you appreciate about their approach?

In what ways can your healthcare professionals further support you?
